# Fused feature signatures to probe tumour radiogenomics relationships

**DOI:** 10.1038/s41598-022-06085-y

**Published:** 2022-02-09

**Authors:** Tian Xia, Ashnil Kumar, Michael Fulham, Dagan Feng, Yue Wang, Eun Young Kim, Younhyun Jung, Jinman Kim

**Affiliations:** 1 School of Computer Science, Faculty of Engineering, The University of Sydney, Sydney, NSW 2006 Australia; 2 School of Biomedical Engineering, Faculty of Engineering, The University of Sydney, Sydney, NSW 2006 Australia; 3 Department of Radiology, Gil Medical Center, Gachon University College of Medicine, Incheon, Republic of Korea; 4 School of Computing, Gachon University, Seongnam, Republic of Korea; 5 Department of Molecular Imaging, Royal Prince Alfred Hospital, Camperdown, NSW 2050 Australia; 6 Department of Electrical and Computer Engineering, Virginia Polytechnic Institute and State University, Arlington, VA 22203 USA

**Keywords:** Cancer genomics, Cancer imaging

## Abstract

Radiogenomics relationships (RRs) aims to identify statistically significant correlations between medical image features and molecular characteristics from analysing tissue samples. Previous radiogenomics studies mainly relied on a single category of image feature extraction techniques (ETs); these are (i) handcrafted ETs that encompass visual imaging characteristics, curated from knowledge of human experts and, (ii) deep ETs that quantify abstract-level imaging characteristics from large data. Prior studies therefore failed to leverage the complementary information that are accessible from fusing the ETs. In this study, we propose a fused feature signature (FF_Sig_): a selection of image features from handcrafted and deep ETs (e.g., transfer learning and fine-tuning of deep learning models). We evaluated the FF_Sig_’s ability to better represent RRs compared to individual ET approaches with two public datasets: the first dataset was used to build the FF_Sig_ using 89 patients with non-small cell lung cancer (NSCLC) comprising of gene expression data and CT images of the thorax and the upper abdomen for each patient; the second NSCLC dataset comprising of 117 patients with CT images and RNA-Seq data and was used as the validation set. Our results show that our FF_Sig_ encoded complementary imaging characteristics of tumours and identified more RRs with a broader range of genes that are related to important biological functions such as tumourigenesis. We suggest that the FF_Sig_ has the potential to identify important RRs that may assist cancer diagnosis and treatment in the future.

## Introduction

Lung cancer is one of the leading causes of cancer death among men and women worldwide. Non-small cell lung cancer (NSCLC) accounts for approximately 85% of all cases of lung cancer^1^. NSCLC diagnosed at an early stage has a 5-year survival rate of up to 80% for small and localised tumours (stage IA)^2^. When compared to patients in advanced stage of NSCLC (stage IV), the 5-year survival rate is 2%^2^.

Advances in the understanding of molecular characteristics of NSCLC have provided insights into the biology of NSCLC and assisted in more precise treatment^3,4^. The usual approach for molecular characterisation is with large-scale gene expression profiling, a technique that determines the process by which information from a gene is converted into a functional gene product, such as proteins. Gene expression analysis at different levels of transcription can provide a global picture of different biological functions and can be identified using computational and statistical methods^5^. Gene expression analysis provided insights that facilitated the development of therapies that target specific biological pathways such as epidermal growth factor receptor (EGFR) in NSCLC that have improved clinical outcomes^6,7^. Cetuximab^8^ is an example of target therapy medications that downregulates the EGFR. Specific types of EGFR mutations, such as the exon 19 deletions and the L858R point mutation are particularly responsive to gefitinib^9^ and erlotinib^10^. These medications are small-molecule tyrosine kinase inhibitors (TKIs) that restrict EGFR from transmitting cellular signals that are related to tumour progression^11^.

Gene expression profiling, however, requires adequate tumour tissue samples that are obtained from core biopsies that sample only a part of the tumour and is invasive and expensive. In contrast, medical imaging is a non-invasive technique that plays a crucial role in routine clinical practice by capturing important imaging visual characteristics. These characteristics are known as image features and can describe tumour’s size, sphericity and, location^12^. Computerised medical image analysis has enabled the high-throughput and quantitative extraction of image features that can capture imaging features that are not quantifiable by visual assessment alone. Previous works showed that image features derived from tumours can predict tumour prognosis and treatment responses for NSCLC^13,14^. These findings contributed to ‘radiogenomics’, a growing research field where the aim is to investigate the relationships between medical imaging features and molecular characteristics. Radiogenomics presents opportunities for the non-invasive assessment of important molecular characteristics that contribute to tumour development. Radiogenomics relationships (RRs) can be determined by identifying statistically significant correlations between image features and gene expressions^15,16^. Another approach to determine RRs involves the use of functional enrichment analysis, such as gene sets enrichment analysis (GSEA)^5^, which uses statistical approaches to associate image features with functions of genetic products. These functions of genetic products are frequently described by using Gene Ontology (GO) terms, which is a formal representation that describes the biology domain with respect to three aspects: molecular functions, cellular components, and biological processes^17^. Published studies showed that RRs may predict the mutation status of key genetic biomarkers in NSCLC such as EGFR and KRAS^18,19^. These biomarkers have been shown to have important implications for the treatment of NSCLC^20^.

There have been attempts at using various image feature extraction techniques (ETs) to determine RRs, for example, between computed tomography (CT) images and tumour prognosis^15^. These radiogenomic studies typically employ a category of ETs that are based on the statistical analysis and medical knowledge of human experts. These ETs quantify ‘handcrafted (HC)’ features that quantify (i) “semantic features” that describe tumour’s visual characteristics, including tumour shape, size, necrosis and contextual information, such as tumour’s surrounding structures and; (ii) “agnostic features” that quantify statistical information of linear relationships about the pixels of the image that human observers consider important^16^ (e.g., colour histograms, Haralick textures and wavelet features^21,22^). Handcrafted ETs have been extensively used to predict mutation status^23^, model cancer outcomes^24^, and response to therapy^13,15^. However, handcrafted ETs are restricted to the human understanding of the disease and prespecified imaging representations. Furthermore, they may not be able to quantify the complex patterns of tumour imaging characteristics and may limit the potential of determining RRs.

Advances in machine learning algorithms are now enabling data-driven approaches for quantifying medical image’s visual characteristics that can complement HC approaches. Deep learning is a method of machine learning that uses techniques such as Convolutional Neural Networks (CNNs) to learn sophisticated abstract and complex imaging characteristics directly from a large volume of labelled training image data^25^. The use of CNNs has achieved state-of-the-art performances in a number of automated medical image analysis tasks that rely on visual characteristics, including tumour classification, detection and, segmentation^26,27^. The data-driven nature of deep learning means that they may be less susceptible to the subjectivity of a human interpreter^25^. Deep learning gave rise to other category of ETs, termed deep ETs, that can quantify intricate complex information from large training data sets, allowing the possibility to detect subtle variations in images of different diseases. The employment of deep ETs in radiogenomics, however, requires large volumes of labelled training data^28^. The quantity of labelled data in medical domains are limited and present a challenge for CNNs to learn comprehensively. Transfer learning (TL) is often used in these circumstances for its ability to leverage CNNs that were pre-trained on large well-labelled natural image (photography) datasets. TL allows the pre-trained CNNs to learn image’s visual characteristics that encode generic visual representations from the natural image datasets^29^; these representations can then be employed in the medical domain to extract image features from smaller medical dataset for radiogenomics. Image features that are quantified using TL-based deep ETs are termed TL features. Since TL does not learn from any medical dataset, they may not necessarily encode specific imaging characteristics of medical images and the different diseases represented within, and hence may be suboptimal for constructing radiogenomic associations. Fine-tuning (FT) is a type of TL technique that uses backpropagation to refine the TL CNN weights through further training on smaller, directly relevant, dataset. This adapts the pre-trained TL CNN to the small relevant dataset without requiring a large well-labelled dataset. Image features that are quantified using FT-based deep ETs are termed FT features. FT has been applied in a range of different medical image analysis tasks such as ultrasound anatomy identification^30^ and lung abnormality detection^31^, and has been used in a study to distinguish the molecular subtype in breast cancer^32^.

Image feature ensemble algorithms offer the opportunity to leverage handcrafted and deep ETs to extract complementary visual characteristics and provide additional information for medical image analysis. Feature fusion is a common ensemble technique that integrates both categories of ETs to produce a more comprehensive image representation of the problem^33^. There have been applications of feature fusion to improve in a range of medical image analysis tasks. Kooi et al.^34^ proposed a computer-aided detection system for mammography by using handcrafted and deep ETs to quantify image features. The deep features were found to be prone to misclassifying benign abnormalities as tumours because both share similar visual characteristics. In their study, handcrafted ETs complemented deep ETs by introducing information that is more difficult for deep features to learn, such as the location and surrounding structures of tumours, thereby increasing the detection performance when compared with using a single category of ETs. Hagerty et al.^35^ demonstrated that using both categories of ETs to quantify image features improved melanoma classification with increased area under the curve (AUC) of receiver operator characteristics (RUC). The handcrafted ETs quantified medically meaningful image features such as lesion colour distribution and atypical pigment network and were complementary to deep ETs that quantified the low-level descriptive image features. Although these ensemble methods demonstrate notable advantages, to the best of our knowledge, the ensemble feature method has yet been investigated for radiogenomics analysis.

In this study, we propose a fused feature signature (FF_Sig_), which is a selection of image features from both HC and deep ETs to encode complementary tumour imaging visual characteristics. We hypothesise that FF_Sig_ can identify more and exclusive RRs when compared to the use of a single category of ETs.

## Methods

### NSCLC–Radiomics–Genomics dataset

We used the public NSCLC Radiomics–Genomics dataset^36^ from the Harvard University, and we refer to this dataset as the ‘NRG-H’. The dataset was sourced from the Cancer Imaging Archive (TCIA)^37^. The NRG-H is a pre-processed and de-identified dataset. The creator of the dataset has indicated that the collection and processing of the dataset were conducted according to national laws and guidelines and approved by the appropriate local trial committee at Maastricht University Medical Center (MUMC1), Maastricht, The Netherlands. The dataset comprises 89 patients (29 W, 60 M; age range 37–85 years) with histologically confirmed NSCLC with T stage (T1–T4)^38^. A detailed dataset description is presented in Supplementary Table S2.

All patients had a CT scan of the thorax/upper abdomen. CT scan slice thickness was between 1.5 and 5 mm. Gene expression information was acquired using the Rosetta/Merck human RSTA custom Affymetrix 2.0 microarray (Affymetrix HuRSTA-2a520709). Gene expression values were normalised using the RMA algorithm 5 in Bioconductor. Gene expression information was accessed via the Gene Expression Omnibus (GEO)^39^. The primary tumours were delineated by an experienced medical imaging specialist (M.F., more than 20 years of experience), slice-by-slice, on trans-axial image slices using open source software (Medical imaging Interaction Toolkit (MITK); version 2016.11^40^). We excluded three patients (all men) because there were lung collapses distal to a proximal tumour and the extent of the tumour could not be reliably identified. Delineations were independently validated by a second clinician (E.K., 7 years of experience). Details of the delineation validation process are described in Supplementary Material Section 1. The annotation differences between the two clinicians are shown in Table S1 in the Supplementary Materials.

### NSCLC-RADIOGENOMICS dataset

The NSCLC-Radiogenomics dataset reported by Bakr et al.^41^ from the Stanford University is a pre-processed and de-identified dataset, and we refer to this dataset as ‘NRG-S’. The creator of the dataset has indicated that the collection and processing of the dataset were conducted under IRB approval from Stanford University and the Veterans Administration Palo Alto Health Care System. The NRG-S dataset comprises CT images and RNA-Seq data from 117 subjects (29 W, 88 M; age range 46–85 years) with histologically confirmed NSCLC with T stage (Tis, T1–T4). A detailed dataset description is presented in Supplementary Table S3.

All patients had a CT scan from the apex of the lung to the adrenal gland in supine position. CT scan thickness was between 0.625 and 3 mm. Detailed scanning parameters, such as the manufacturer attributes are specified in the DICOM headers. Total RNA was extracted from the tissue and analysed with RNA sequencing technology. Gene expression information was processed using the STAR algorithm^42^ and Cufflinks version 2.0.2^43^. Gene expression information was accessed via the Gene Expression Omnibus (GEO)^39^. Primary tumours were segmented using an unpublished automatic segmentation algorithm on the axial image slices for all 117 subjects. Segmentations were viewed by a thoracic radiologist (M.K.) with more than 5 years of experience and edited as necessary using ePAD. An additional thoracic radiologist (A.N.L.) reviewed and approved the final segmentations.

### Experimental overview

**Figure 1:**
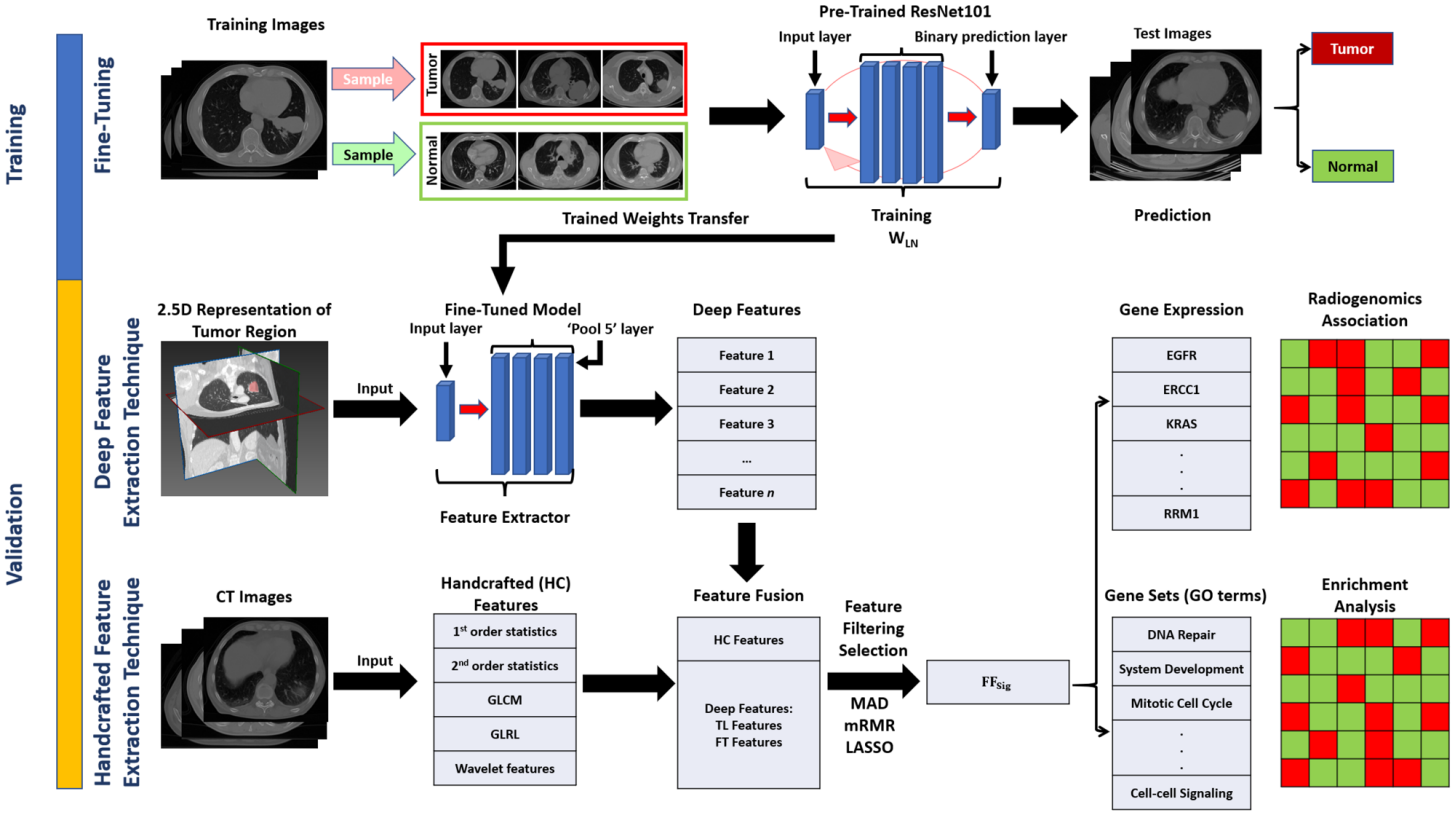
The workflow for generating the FF_Sig_ and the identification of RRs with genes and GO terms. The workflow was implemented using the NRG-H dataset and validated on the NRG-S dataset.

An overview of the experimental design is outlined in Fig. 1. HC and deep ETs are used to extract HC, TL and FT feature from delineated tumour ROIs from CT image volumes. HC features are extracted from the CT image volume directly. FT features are extracted from a 2.5D representation of the CT data around the tumour centroid^31^. The extracted HC, TL and FT features are fused into a feature matrix using concatenation. The FF_Sig_ is generated by applying a multi-step feature selection procedure involving median absolute deviation (MAD), minimum redundancy maximum relevance (mRMR), and least absolute shrinkage and selection operator (LASSO) generalised linear model. RRs are determined by using Spearman rank correlation between FF_Sig_ and the averaged gene expressions. RRs between image features signatures and GO terms are determined by using GSEA. For evaluation purposes, the same multi-step feature selection procedure is applied to HC, TL and FT features. The resulting collections of image features are denoted as HC_Sig_, TL_Sig_ and FT_Sig_, respectively. The training of the deep ETs was performed on the NRG-H dataset; the ETs were then used to extract image features and generate FF_Sig_. We validated the robustness and generalisability of the FF_Sig_ by applying NRG-H trained deep ETs to the validation NRG-S dataset.

### Image features

#### HC and deep ETs

We employed a set of standard HC ETs that are implemented in the pyradiomics framework to quantify HC features^15,44^. For each patient, we extracted a well-documented set of 431 HC features from CT volumes^45,46^. These 431 HC features comprised the following: (a) first-order statistics, describing the distribution of voxel intensities; (b) shape and size that are geometric descriptors of tumoural 3D characteristics such as compactness and surface area; (c) textural or co-occurrence matrix features to illustrate the spatial distribution of the voxel intensities and, (d) first order statistics and textural features of the wavelet decompositions of the raw imaging data. The detailed description to the 431 HC features is provided in Supplementary Material Section 4.

Deep ETs used a ResNet-101 backbone that was pre-trained on ImageNet ILSVRC challenge data. ResNet-101 is a well-established CNN architecture, which introduced the concept of ‘residual blocks’, a combination of skip connections and identity mapping to learn deeper features, and is robust to accuracy degradation^47^. ResNet-101 is a robust and efficient CNN architecture that have been applied in a range of different medical image analysis tasks such as brain tumour classification^48^ and segmentation for kidney and space-occupying lesion area^49^. In comparison with other widely used pre-trained deep models, such as GoogLeNet^50^, ResNet-101 has demonstrated superior performance in natural image detection across different datasets such as PASCAL VOC 2012 and ImageNet detection^28,50^. The pre-training of ResNet-101 on ImageNet ILSVRC challenge data (millions of well-annotated images that belongs to 1000 natural object classes) allowed it to extract deep features that represent generic image characteristics applicable to all images such as edge and texture^29^, which has been demonstrated to be useful descriptors for medical images^47^. We have compared the tumour classification performance between ResNet-101 and some of the most commonly used pre-trained deep models such as VGG-19^51^ on the testing set of the NRG-H. The detailed protocol for evaluating the tumour classification performance between the deep models is presented in Supplementary Materials Section 2.

To adopt the ResNet-101 model with pre-trained weight and to recognise the features in the NSCLC CT data, we fine-tuned it for the task of identifying CT images that contained tumours. The 86 subjects from the NRG-H dataset were divided into two groups: a training set that comprises imaging data from 69 patients and, a testing set that comprises imaging data from 17 patients. Subjects in the training and testing groups were randomly selected. We implemented a fivefold cross-validation strategy on the training set of 69 patients to fine-tune the ResNet-101 model. The testing set of 17 subjects was ‘held out’/’unseen’ during the fine-tuning process and serves to assess the robustness and generalisability of those fine-tuned ResNet-101 models.

From the training set, a total of 2420 CT image slices were sampled for the fine-tuning task. The CT images from the training patients were augmented to avoid overfitting during the fine-tuning. Training data was augmented by randomly rotating images between 0 to 360 degrees and translating the rotated images between − 5 to 5 pixels on both the x and y-axis. The last layer of the pre-trained ResNet-101 was replaced by a new fully connected layer to accommodate the classification task. The weight learn rate factor and bias learn rate factor were set to 20 for the new fully connected layer.

The fine-tuning process of the ResNet-101 model involved 300 epochs of training using stochastic gradient descent with a momentum of 0.9 and a batch size of 5. The Learning rate was set at 1 × 10^–3^, with L2Regularization set at 0.001. For every 100 epochs, the learning rate decreased by the factor of 0.1. These hyperparameters were determined and tuned by using the widely adopted random search optimisation method^52^. This is achieved by finding the optimum model which consists of the combination of hyperparameters that give the best overall performance for the classification task. Fine-tuning was implemented using MATLAB 2019b on a machine running Ubuntu 18.04, with an 11 GB NVIDIA RTX 2080 Ti GPU and CUDA 10.1. The fine-tuned model with the best overall performance was selected to serve as deep ET for FT features (Supplementary Table S4). The performance of the selected deep ET was then assessed on the second NRG-S dataset (Supplementary Table S5).

#### Image feature extraction

HC features were extracted directly from the volumetric CT images using the pyradiomics framework. TL and FT features were extracted from the ‘pool5’ layer of the ResNet-101 model. We used the axial, sagittal and coronal views of the tumour ROI from the volumetric CT images as the input for deep ETs^31^. All views were centred on the physical centroid of the tumour ROI. Such an aggregated views display a 2.5-dimensional (2.5D) representation of the tumour ROI^53^. The 2.5D representation for deep ETs contains richer spatial information of neighbouring pixels compared with traditional 2D images while demanding less computational power when compared with running ETs directly on 3D image volumes^54^. For each view of the 2.5D representation, gray values were normalised from [0, 4096] to [0, 255] using a linear transformation. All three input slices were resized to 224 × 224 to fit the input size of ResNet-101 using nearest-neighbour interpolation and were padded with zeros to preserve the tumour aspect ratio. 6144 FT and 6144 TL were extracted from each CT image.

#### Image feature fusion and selection

We used a feature fusion strategy that concatenates the HC, TL and FT feature together to generate a feature matrix across the patients^55^. The 431 HC, 6144 TL, and 6144 FT features were then fused into a single 12,719-dimensional feature matrix using concatenation. The resulting high-dimensional feature matrix presented challenges in performing statistically significant analyses as the number of features is much larger than observations^56,57^. In such circumstances, small random fluctuations in individual features may be mistaken for important variance in the data and lead to the selection of features that are suboptimal for representing the observations. In addition, the concatenation may cause redundant features from individual extraction techniques to be contained within the matrix and add complexity during data interpretation. Feature selection is a technique to reduce the dimensionality and identify the subset of optimal and robust features that provide the best predictive power^58^. We hence applied a multi-step image feature selection scheme that aims to: (i) reduce the dimensionality of the concatenated feature matrix; (ii) remove image features that are redundant or irrelevant to the histology classification of tumours; and (iii) identify a set of image features that are most relevant to the histology characteristics of patients.

The reduction of the dimensionality removed features that have poor variability and dispersion across patients. These features do not reflect the variances in tumour imaging characteristics and therefore unideal for identifying radiogenomics associations. We used the median absolute deviation (MAD) as an indication for these features as it measures the variability across features and is robust against outliers in the concatenated feature matrix.

The second stage reduced the dimensionality of the remaining features by removing those that are redundant or irrelevant to the histology characteristics of patients. The histology characterisation is a crucial parameter that indicates the subtypes of the disease and may also contain information that reflects distinct patterns of genetic alterations^59^. The removal of biologically irrelevant and redundant features, therefore, prevents the discovery of meaningless radiogenomics associations. The histology characteristics of each patient were categorised into one the following classes: (1) squamous cell carcinoma, (2) adenocarcinoma and (3) other types including Non-Small cell and Not otherwise specified (NOS).

We used mRMR, a widely-adopted approach for feature selection, to produce a subset of features with high biological relevance^60^. The mRMR method selects features that have: (i) the maximal mutual information between the total feature set and the histology characterisation and (ii) the minimal mutual information between the selected features subset and the total feature set. A total of 100 features were selected using the mRMR method, taking into consideration of the number of patients as well as the original dimensionality of the feature matrix^61^.

The last stage of feature selection employed LASSO regularisation for generalised linear models to identify the set of remaining image features that are most relevant to the histology characteristics of patients. LASSO shrinks regression coefficients towards zero-based on regularisation weight λ; features with non-coefficients are those that are related to predicting histological characteristics and hence are selected. We performed 10-fold cross-validation to identify the value of λ with the minimum cross-validation error. The outcome of this stage was the final FF_Sig_ that was used for identifying radiogenomics associations with gene expressions and GO terms. We also applied the multi-stage image feature selection process to the HC, TL and FT features individually for comparison. The resulting image feature signatures are hereafter denoted as ‘HC_Sig_’, ‘TL_Sig_’ and ‘FT_Sig_’, correspondingly.

### Associating FF_Sig_ with primary tumour T stages

The tumour, node, metastasis (TNM) staging is the most important clinical parameter to predict survival and establish treatment plans^62^. The T stage describes the size of the primary tumours and their involvement in the adjacent structures. We investigate investigated if the FF_Sig_ is relevant to primary tumour T stages (T1–T4) prior to the radiogenomics analysis. We used unsupervised k-means clustering to the FF_Sig_ to stratify the patients into distinct groups; the patient clusters were defined using 10 repeated new initial cluster centroid positions with a maximum of 1000 iterations. We compared the three patient clusters with the distribution of the T stage. We used the χ^2^ test of independence to assess the ability of the FF_Sig_ to encode tumour staging characteristics^63^. For comparative evaluation, the HC_Sig_, TL_Sig_ and FT_Sig_ were also validated for their relevance to the T stage.

### Functional gene analysis

#### Gene selection

Probes that map to multiple unique gene symbols were discarded and the repeated total gene expression values of the same gene were averaged. Gene expression data may contain redundant genes that are irrelevant to the disease. We used the following process to remove genes that had low variance, entropy and absolute expression value because such genes showed poor variability and dispersion, and therefore may not reflect the differences in the underlying tumour biology. We firstly removed genes with a variance of less than one-quarter percentile, as such genes may not reflect changes in tumour biological behaviours. The averaged gene expression was filtered to remove the genes with a variance of less than one-quarter percentile across all patients. The remaining genes were then filtered to remove genes that have an absolute expression level in the lowest quarter percentile of the gene expression; genes with low absolute expression were removed because they are prone to errors due to large quantisation or spot hybridisation. Finally, gene expressions were filtered to remove the genes with an entropy value that is less than the quarter percentile; genes with low entropy are considered to be consistently expressed across patients and may not reflect the variance in tumour biological characteristics^64^.

#### Radiogenomics analysis

We determined RRs between the FF_Sig_ with the averaged gene expressions using the Spearman rank correlation. We also employed functional enrichment analysis to enrich radiogenomics relationships with GO terms. We used 1046 gene sets from the C5 collection of MSigDB^65^, which categorise the following GO terms: molecular function, cellular component and biological process. The gene list was generated by ranking the radiogenomics associations for each of the features from FF_Sig_ in descending order. Gene sets that include between 15 and 500 contributing genes were selected for the enrichment analysis as was the standard protocol in prior work^15^. The determined RRs were then assessed using a pre-ranked functional enrichment analysis. In this process, the radiogenomics relationships between FF_Sig_ and gene expressions were sorted to provide a ranked gene list based on the strength of the Spearman rank correlation.

We used the pre-ranked gene list to perform GSEA, which derives the association between the provided ranked gene list and GO terms by testing the enrichment of each annotated term iteratively in a linear model. The enriched radiogenomics relationships with GO terms can be quantified by calculating normalised enrichment scores (NES) based on the number of genes. NES indicates the degree to which a GO term is overrepresented by the radiogenomics relationships. To ensure that only significantly associated genes were used for functional enrichment analysis, RRs with p-value < 0.001 were selected and ranked and serve as input to the functional enrichment analysis with GO terms. The same procedure was applied to the HC_Sig_, TL_Sig_ and FT_Sig_ for comparative experiments.

### Evaluation strategy

We evaluated the performance of FF_Sig_ by: (i) determining if the proposed FF_Sig_ can encode complementary medical image visual characteristics when compared with other image feature signatures; (ii) determining if the proposed FF_Sig_ is relevant to the tumour T stage by using the χ^2^ test of independence; (iii) assessing the distribution of RRs with genes; (iv) assessing the distribution of RRs with GO terms; (v) determining if the proposed FF_Sig_ can identify exclusive RRs with genetic biomarkers of NSCLC and GO terms that are related to NSCLC.

## Results

### Image feature signatures

After performing the multi-stage image feature fusion and selection on the NRG-H dataset, all four feature signatures were generated. FF_Sig_ is comprised of features that were all extracted from sagittal planes of the 2.5D presentation and has the highest number of features at 7. TL_Sig_ is also comprised of features that were all extracted from sagittal planes and has 6 features. FT_Sig_ is comprised of features that were extracted from 1 axial and 2 sagittal planes and has 3 features. HC_Sig_ is comprised of features that were extracted directly from image volumes and have 2 features. In contrast, our validation experiments on the NRG-S dataset show that only FF_Sig_, FT_Sig_ and HC_Sig_ were generated after performing the multi-stage image feature fusion and selection. Our validation results from the NRG-S dataset show that the FF_Sig_ is comprised of features that were all extracted from 1 axial and 12 sagittal planes of the 2.5D presentation and has the highest number of features at 13. TL_Sig_ was not generated as none of the TL features were selected after the multi-step feature selection scheme. FT_Sig_ is comprised of 2 image features that were extracted from axial planes. HC_Sig_ is comprised of features that were extracted directly from image volumes and have 1 feature only.

### Image signatures and T stage

**Figure 2:**
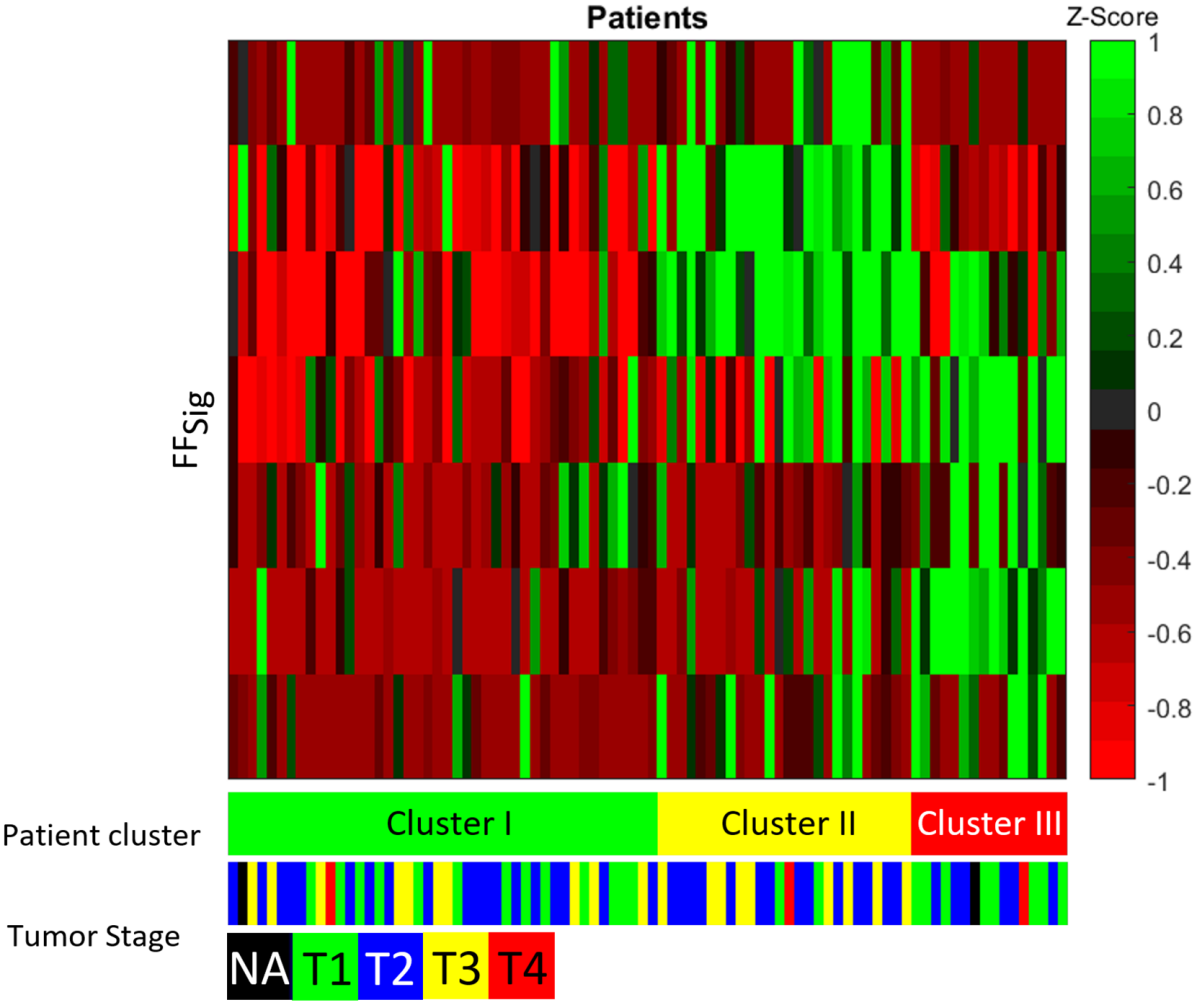
Heatmap of the FF_Sig_ across patient clusters with corresponding T stage from the NRG-H dataset. The heatmap was generated using MATLAB, version 2019b, URL: https://www.mathworks.com/products/matlab.html.

In our experiment on the NRG-H dataset, the HC, TL and TF features were significantly associated with the T stage parameters (T1–T4) across patient clusters. The χ^2^ test statistics for HC, TL and TF features with T stage parameters are p < 2.9 × 10^–4^, p < 5.0 × 10^–3^ and p < 4.8 × 10^–2^, respectively. For image signatures, FF_Sig_ was significantly associated with primary tumour T stages (χ^2^ test, p < 4.0 × 10^–2^). None of the HC_Sig_, TL_Sig_ or FT_Sig_ is found to be significantly associated with primary tumour T stages, their χ^2^ test statistics are p > 0.8, p > 6.0 × 10^–2^ and p > 0.5, respectively. Figure 2 illustrate the relationships among FF_Sig_, T stages and patient clusters from the NRG-H dataset. Each row of the heatmap represents one image feature that comprises the FF_Sig._ Each column of the heatmap represents a single patient. Z-score is calculated for each radiomics feature across patients. Z-score shows the distinct distribution of T stage parameters across patient clusters. The association between FF_Sig_ and the T stage parameters is indicated by the grouped image features among the patient cluster II and III. The distinct pattern is represented using a z-score of image features that were extracted from each patient.

In our validation experiment on the NRG-S dataset, none of the HC, TL and TF features were significantly associated with the T stage parameters (Tis, T1–T4) across patient clusters. The χ^2^ test statistics for HC, TL and TF features with T stage parameters are p > 0.7, p > 0.7 and p > 0.8, respectively. For image signatures, none of the FF_Sig_, HC_Sig_ or FT_Sig_ was found to be significantly associated with primary tumour T stages, their χ^2^ test statistics are p > 0.5, p > 0.5 and p > 0.2, respectively.

### RRs between image feature signatures and genes

**Figure 3:**
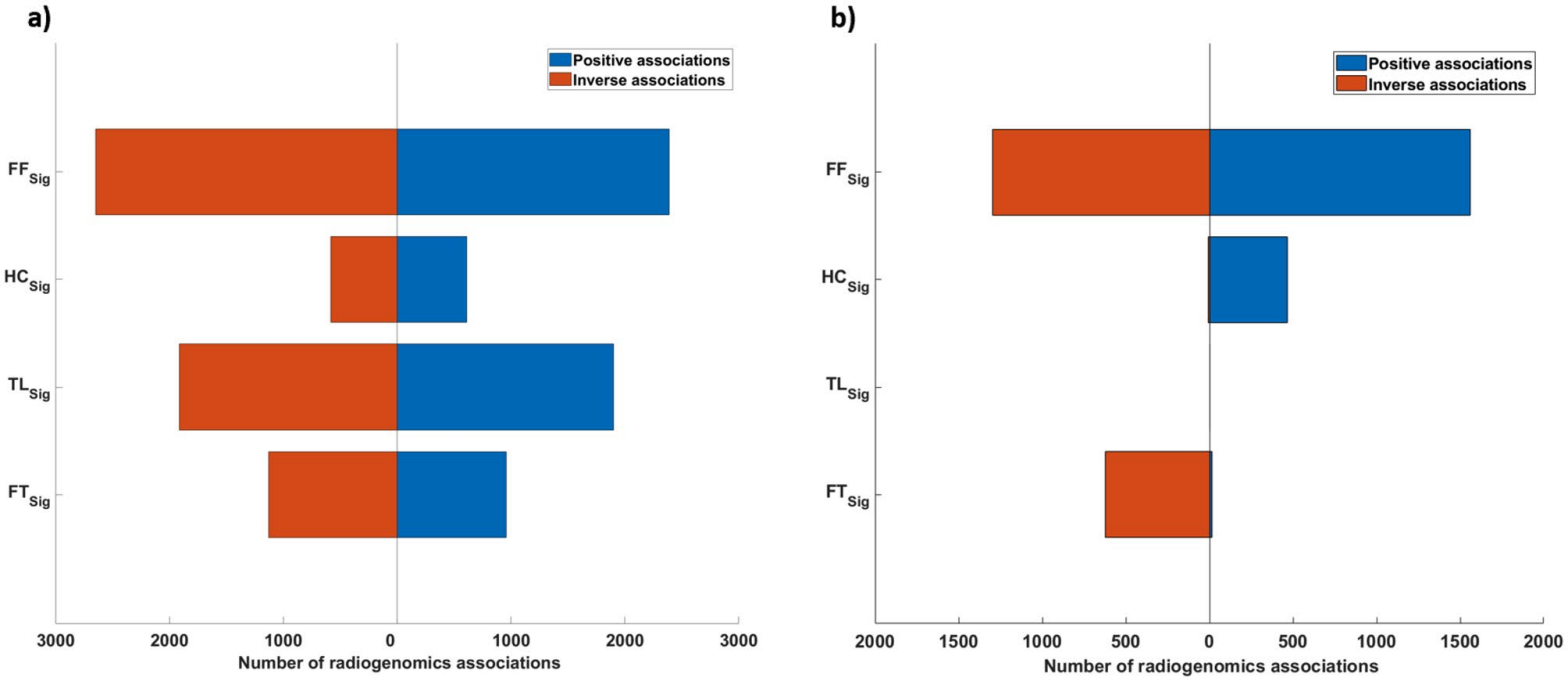
The distribution of RRs between feature signatures and: (**a**) gene expression value of the processed genes (n = 11,318) from the NRG-H dataset. (**b**) Gene expression value of the processed genes (n = 2993) from the NRG-S dataset.

**Figure 4:**
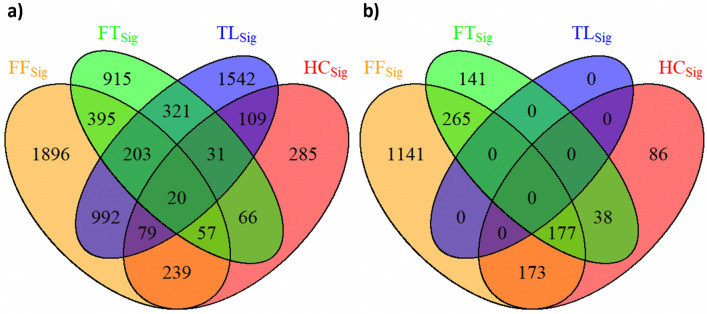
Venn diagram shows the distribution of unique genes that were associated with FF_Sig_, HC_Sig_, TL_Sig_, and FT_Sig_: (**a**) generated using the NRG-H dataset. (**b**) generated using the NRG-S dataset.

After gene expression filtering, a total of 11,318 gene expression remained from the NRG-H dataset to establish radiogenomics associations. Notably, two of the key biomarkers for NSCLC: KRAS and RRM1, were filtered due to low variance across the patients in the NRG-H dataset. Figure 3a represents the distribution of RRs that were determined between the averaged gene expression values of 11,318 individual genes and FF_Sig_, HC_Sig_, TL_Sig_ and FT_Sig_. FF_Sig_ identified the highest number of RRs at 5039 and correlated with the highest number of genes at 3881. HC_Sig_ identified 1193 RRs with 886 genes. TL_Sig_ identified 3816 RRs with 3297 genes. FT_Sig_ identified 2089 RRs with 2008 genes. Figure 4a details the distribution of unique genes that were associated with FF_Sig_, HC_Sig_, TL_Sig_, and FT_Sig_. Among the 3881 unique genes that were associated with the FF_Sig,_ 1896 unique genes cannot be associated with any of the HC_Sig_, TL_Sig_, and FT_Sig_. In contrast, a total number of 3269 unique genes were associated with one of the HC_Sig_, TL_Sig_, and FT_Sig_, but were not correlated with the FF_Sig_.

**Figure 5:**
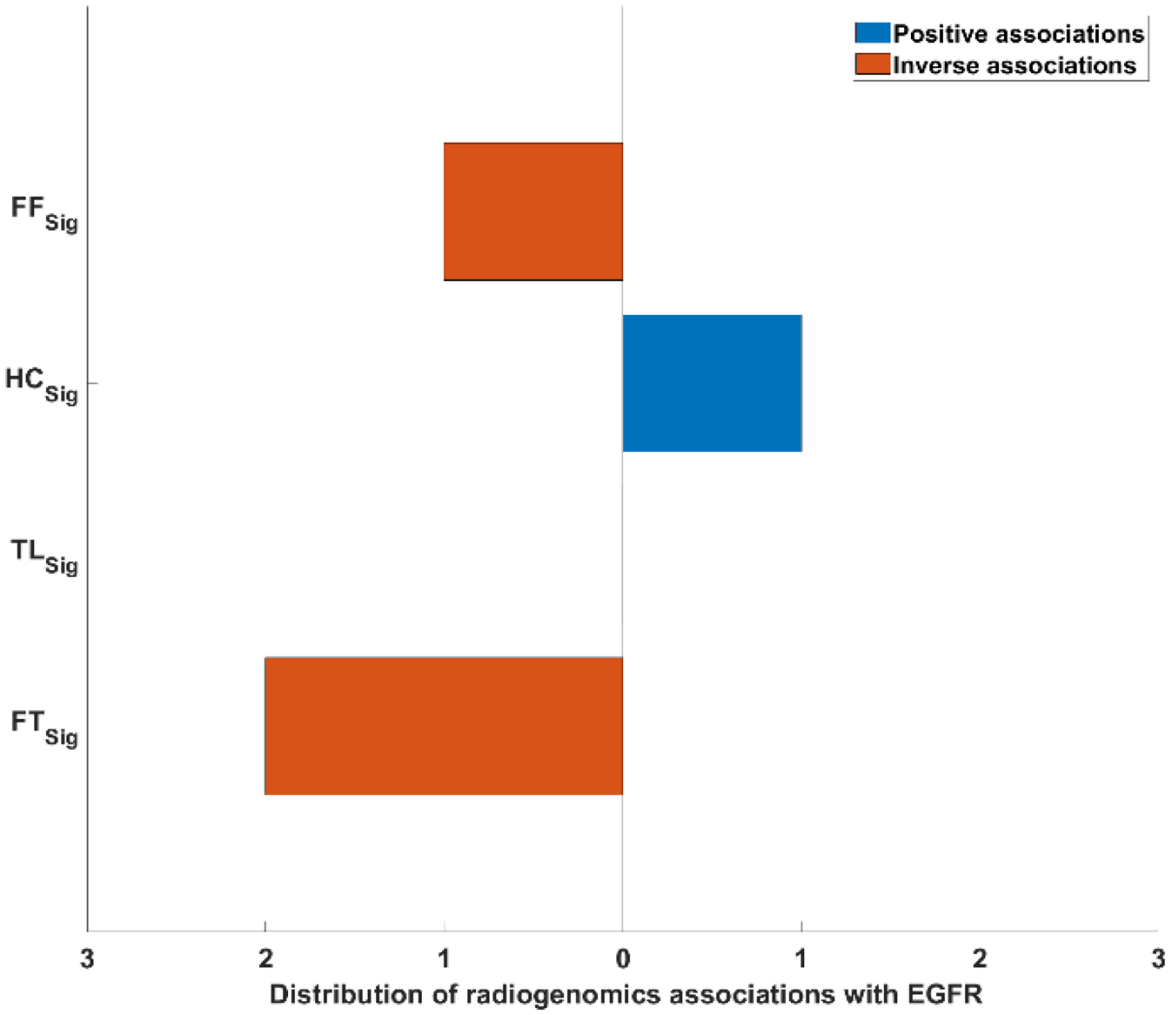
The distribution of RRs between the FF_Sig_ with the key genetic biomarker of EGFR from the NRG-H dataset, in comparison to HC_Sig_, TL_Sig_ and FT_Sig_.

Table 1 compares the strengths of all RRs that were determined using the FF_Sig_ against those determined using HC_Sig_, TL_Sig_ and FT_Sig_. Our results show stronger RRs are identified between the FF_Sig_ and genes, when compared with HC_Sig_ and TL_Sig_, in the inverse direction. The FF_Sig,_ however, did not show stronger inverse RRs when compared with FT_Sig_. On the other hand, the FF_Sig_ did not show stronger positive RRs when compared with HC_Sig_, TL_Sig_ nor FT_Sig_. Figure 5 illustrates the distribution of RRs that were determined between image feature signatures of the FF_Sig_, HC_Sig_, TL_Sig_, FT_Sig_ with the gene expression value from the key genetic biomarkers of EGFR for NSCLC^20^. Our result shows that the FF_Sig_ and FT_Sig_ were inversely correlated with EGFR expression. In contrast, HC_Sig_ is shown to be the only positive RRs with EGFR. Notably, FT_Sig_ shows to derive more and stronger inverse RRs with EGFR when compared with the FF_Sig._ In addition, our result shows that ERCC1, a key genetic biomarker for NSCLC, is exclusively correlated with a single feature from the FF_Sig,_ where the same feature showed inverse RRs with EGFR previously.

**Table 1:** Two-sample *t* tests that assess the strengths of all RRs constructed using the FF_Sig_ with HC_Sig_, TL_Sig_ and FT_Sig_, in both statistical directions on the NRG-H dataset.

Feature signature	**HC**_**Sig**_	**TL**_**Sig**_	**FT**_**Sig**_
**Strength of positive RRs (two-sample *t* test)**			
FF_Sig_	p > 0.2	p > 0.7	p > 0.3
**Strength of inverse RRs (two-sample *t* test)**			
FF_Sig_	p < 1 × 10^–3^	p < 1 × 10^–2^	p > 0.6

The gene selection process was repeated in our validation experiments on the NRG-S dataset. A total of 22,126 unique genes were identified for each patient from the NRG-S dataset. After gene selection, 2993 gene expression remained from the NRG-S dataset to establish radiogenomics associations. In comparison to NRG-H, three of the key biomarkers for NSCLC: EGFR, KRAS and ERCC1, were filtered due to low variance across the patients in the NRG-S dataset. Figure 3b represents the distribution of RRs that were determined between the averaged gene expression values of 2993 individual genes and FF_Sig_, HC_Sig_, and FT_Sig_. Radiogenomics analysis show that FF_Sig_ identified the highest number of RRs at 2856 and correlated with the highest number of genes at 1756. HC_Sig_ identified 474 RRs with 474 genes. FT_Sig_ identified 642 RRs with 621 genes. In addition, our result shows that RRM1, a key genetic biomarker for NSCLC, is exclusively correlated with a single feature from the FF_Sig_. Figure 4b details the distribution of unique genes that were associated with FF_Sig_, HC_Sig_, TL_Sig_, and FT_Sig_. Among the 1756 unique genes that were associated with the FF_Sig_, 1141 unique genes cannot be associated with any of the HC_Sig_ and FT_Sig_. In contrast, a total number of 265 unique genes were associated with one of the HC_Sig_ and FT_Sig_ but were not correlated with the FF_Sig_. Table 2 compares the strengths of all RRs that were determined using the FF_Sig_ against those determined using HC_Sig_, and FT_Sig_. Our validation results show that the FF_Sig_ did not identify stronger RRs with genes, when compared with HC_Sig_ and TL_Sig_, in both statistical directions.

**Table 2:** Two-sample *t* tests that assess the strengths of all RRs constructed using the FF_Sig_ with HC_Sig_ and FT_Sig_, in both statistical directions on the NRG-S dataset.

Feature signature	HC_Sig_	FT_Sig_
**Strength of positive RRs (two-sample *t* test)**		
FF_Sig_	p > 0.8	p > 0.2
**Strength of inverse RRs (two-sample *t* test)**		
FF_Sig_	p > 0.3	p > 0.08

### RRs between image feature signatures and GO terms

**Figure 6:**
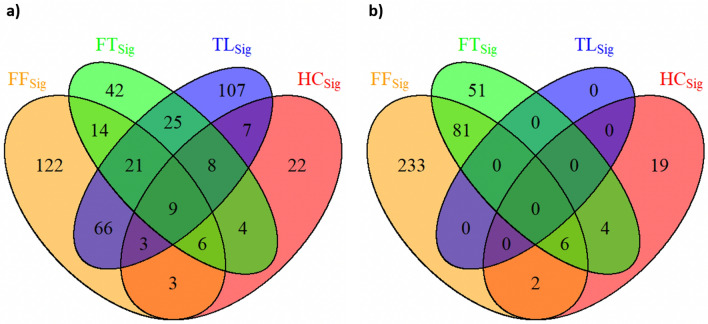
Venn diagram shows the distribution of GO terms that were associated with image feature signatures of FF_Sig_, TL_Sig_, FT_Sig_ and HC_Sig_: (**a**) generated using the NRG-H dataset. (**b**) Generated using the NRG-S dataset.

From our experiments using the NRG-H dataset, FF_Sig_ determined RRs with the highest number of GO terms at 244. HC_Sig_ determined RRs with 62 GO terms TL_Sig_ determined RRs with 246 GO terms. FT_Sig_ determined RRs with 129 GO terms. Figure 6a details the distribution of GO terms that were associated with image feature signatures of FF_Sig,_ HC_Sig_, TL_Sig_, and FT_Sig_. Among the 244 GO terms that have RRs with by FF_Sig,_ 122 GO terms were exclusively enriched; these GO terms account for 50% of the total enriched GO terms or 13.8% of the total 1046 GO terms.

Table 3 shows the GO terms with the highest NES. Notably, FF_Sig_ determined RRs with GO terms that exhibit distinct patterns relating to the biological functions and cellular behaviours: (i) 3 GO terms were related to lumen structures including organelle, nuclear and membrane; (ii) 2 GO terms were reflecting biosynthesis processes that involve glycoprotein or macromolecule; (iii) 3 GO terms were related to the response mechanism to viruses, other organism or biotic stimulus, and other types of stimulus processes. In comparison, our results also show that TL_Sig_ determined RRs with 4 GO terms that are associated with fraction activities. In addition, FT_Sig_ determined RRs with GO terms that are related to enzyme activities. In contrast, HC_Sig_ determined RRs with GO terms are shown to be without overlaps in their biological functionalities.

**Table 3:** The GO terms that have RRs with FF_Sig_, HC_Sig_, TL_Sig_ and FT_Sig_ with positive and negative associations from the NRG-H dataset.

	NES		NES
**FF**_**Sig**_		**HC**_**Sig**_	
Organelle lumen	2.43	Extracellular region	1.74
Nuclear lumen	2.22	Regulation of transferase activity	0.60
Membrane enclosed lumen	2.19	Transferase activity transferring phosphorus containing groups	0.58
Glycoprotein biosynthetic process	1.98	Protein kinase activity	0.58
Macromolecule biosynthetic process	1.94	Stress activated protein kinase signalling pathway	0.58
Response to virus	− 1.98	Carbohydrate metabolic process	− 0.99
Cell cell signaling	− 1.98	Phosphoric monoester hydrolase activity	− 0.99
Response to other organism	− 2.00	Phosphoric ester hydrolase activity	− 1.01
Anatomical structure morphogenesis	− 2.01	Alcohol metabolic process	− 1.02
Response to biotic stimulus	− 2.01	Hydrolase activity acting on ester bonds	− 1.02
**TL**_**Sig**_		**FT**_**Sig**_	
Cell fraction	2.17	Anatomical structure morphogenesis	1.85
Membrane fraction	2.03	Enzyme regulator activity	1.80
Phosphoric ester hydrolase activity	2.02	Enzyme activator activity	1.79
Soluble fraction	1.96	Enzyme linked receptor protein signalling pathway	1.77
Insoluble fraction	1.96	Membrane fraction	1.73
Homophilic cell adhesion	− 1.66	Extracellular region part	− 1.23
Sulfuric ester hydrolase activity	− 1.67	Extracellular space	− 1.23
Nervous system development	− 1.68	Phosphorylation	− 1.24
Regulation of anatomical structure morphogenesis	− 1.68	Lipase activity	− 1.25
Cell surface	− 1.99	Female pregnancy	− 1.27

Table 4 shows the comparison between GO terms that have exclusive RRs with FF_Sig_ and those GO terms that are restricted to have RRs with FF_Sig_. Among the GO terms with the highest NES, our result shows clusters of biological functions and cellular behaviours that have exclusive RRs with the FF_Sig_: (i) 3 GO terms were related to kinase activities for transmembrane receptor protein and tyrosine kinase; (ii) 2 GO terms were related to metabolism activities; The identical 3 GO terms were most enriched by FF_Sig_ and related to the virus response mechanism. In contrast, our result shows 2 groups of related biological functions among the GO terms that were restricted to FF_Sig._ Such GO terms are related to fraction processes and enzyme activities.

**Table 4:** The GO terms that have the highest NES and exclusively RRs with FF_Sig_ (left) and the GO terms that are restricted to have RRs with FF_Sig_ (right), experimented on the NRG-H dataset.

FF_Sig_ exclusive	NES	FF_Sig_ restricted	NES
Transmembrane receptor protein kinase activity	1.61	Soluble fraction	1.96
Protein tyrosine kinase activity	1.60	Insoluble fraction	1.96
Transmembrane receptor protein tyrosine kinase activity	1.53	Enzyme regulator activity	1.80
Generation of precursor metabolic and energy	1.47	Enzyme activator activity	1.79
Phospholipid metabolic process	1.42	Molecular adaptor activity	1.73
RNA processing	− 1.85	Generation of neurons	− 1.66
Organ morphogenesis	− 1.95	Homophilic cell adhesion	− 1.67
Response to virus	− 1.98	Sulfuric ester hydrolase activity	− 1.67
Response to other organism	− 2.00	Regulation of anatomical structure morphogenesis	− 1.68
Response to biotic stimulus	− 2.01	Cell surface	− 1.99

From our validation experiment on the NRG-S dataset, functional gene enrichment analysis reveals that FF_Sig_ determined RRs with the highest number of GO terms at 322. HC_Sig_ determined RRs with 31 GO terms. TL_Sig_ determined RRs with 0 GO terms. Figure 6b details the distribution of GO terms that were associated with image feature signatures of FF_Sig,_ HC_Sig_, TL_Sig_, and FT_Sig_. FT_Sig_ determined RRs with 142 GO terms. Among the 322 GO terms that have RRs with by FF_Sig,_ 233 GO terms were exclusively enriched; these GO terms account for 72.4% of the total enriched GO terms or 22.3% of the total 1046 GO terms.

Table 5 shows the GO terms with the highest NES. Notably, FF_Sig_ determined RRs with GO terms that exhibit distinct patterns relating to the cellular structure: (i) 3 GO terms were related to lumen structures including organelle, nuclear and membrane; (ii) 2 GO terms that reflect the cell junction. In comparison, FT_Sig_ determined RRs with GO terms that are related to cellular structures, protein transportation and localisation. HC_Sig_ determined RRs with GO terms that are related to signalling pathways, such as cAMP mediated signalling and second messenger mediated signalling.

**Table 5:** The GO terms that have RRs with FF_Sig_, HC_Sig_ and FT_Sig_ with positive and negative associations from the NRG-S dataset.

FF_Sig_	NES	HC_Sig_	NES	FT_Sig_	NES
Perinuclear region of cytoplasm	2.62	Sensory perception	1.80	Intracellular protein transport	2.62
Nervous system development	2.58	Monooxygenase activity	1.78	Establishment of protein localisation	2.61
Membrane organisation and biogenesis	2.45	Oxygen binding	1.78	Macromolecule localisation	2.61
Intercellular junction	2.05	Electron transport (GO 0006118)	1.75	Protein localisation	2.54
Tight junction	1.96	Neurological system process	1.70	Protein transport	2.52
Kinase activity	− 2.00	Second messenger mediated signalling	− 0.77	Soluble fraction	− 1.61
Endoplasmic reticulum	− 2.11	Establishment and or maintenance of cell polarity	− 0.77	Organelle lumen	− 1.62
Nuclear lumen	− 2.19	Regulation of catalytic activity	− 0.77	Nucleolus	− 1.65
Organelle lumen	− 2.84	cAMP mediated signalling	− 0.77	Nuclear lumen	− 1.67
Membrane enclosed lumen	− 3.06	G protein signalling adenylate cyclase activating pathway	− 0.77	Membrane enclosed lumen	− 1.71

Table 6 shows the comparison between GO terms that have exclusive RRs with FF_Sig_ and those GO terms that are restricted to have RRs with FF_Sig_. Among the GO terms with the highest NES, our validation results show a cluster of biological functions and cellular behaviours that have exclusive RRs with the FF_Sig_: (i) 3 GO terms were related to peptidase activity; (ii) 2 GO terms that reflect the cell junction. In contrast, our result shows 2 groups of related biological functions among the GO terms that were restricted to FF_Sig._ Such GO terms are related to the intrinsic components of organelle membranes and metabolic processes.

**Table 6:** The GO terms that have the highest NES and exclusively RRs with FF_Sig_ (left) and the GO terms that are restricted to have RRs with FF_Sig_ (right), experimented on the NRG-S dataset.

FF_Sig_ exclusive	NES	FF_Sig_ restricted	NES
Perinuclear region of cytoplasm	2.62	Positive regulation of metabolic process	1.93
Membrane organisation and biogenesis	2.45	Positive regulation of cellular metabolic process	1.90
Intercellular junction	2.05	Neurite development	1.90
Tight junction	1.96	Steroid hormone receptor signalling pathway	1.89
Apical junction complex	1.94	Cellular lipid catabolic process	1.88
Serine type peptidase activity	− 1.57	cAMP mediated signalling	− 0.77
Serine hydrolase activity	− 1.58	G Protein signalling adenylate cyclase activating pathway	− 0.77
Serine type endopeptidase activity	− 1.60	Intrinsic to Golgi membrane	− 0.88
Peptidase activity	− 1.75	Intrinsic to organelle membrane	− 0.93
Endopeptidase activity	− 1.76	Integral to organelle membrane	− 0.93

## Discussion

Our main findings are that our FF_Sig_: (i) encoded complementary medical image’s visual characteristics when compared with other image feature signatures; (ii) determined a greater number of RRs with a greater number of genes; (iii) determined RRs with distinct GO terms; (iv) determined exclusive RRs with genetic biomarkers of NSCLC and GO terms that are related to NSCLC and (v) is robust and generalisable for determining RRs when validated on NRG-S.

From our experiments using the NRG-H dataset, the FF_Sig_ comprises 7 image features that are complementary to image features that were selected in the HC_sig_, TL_sig_, and FT_sig_. Image features that are included in the FF_Sig_ can be traced back to the 6144-dimensional TL features. This finding indicates that the multi-step feature selection scheme prioritised a set of complementary image features that are relevant to the histological characteristics while reducing the overall redundancy in the information captured. This finding suggests that the FF_Sig_ encodes unique medical imaging visual characteristics when compared with other image signatures. The FF_Sig_ was the only feature signature that produced a significant association (p < 0.05) with the T stage. The HC_Sig_, TL_Sig_, and FT_Sig_ did not have any association with the T stage, despite the fact that the FF_Sig_ was selected from the HC, FT, and TL features. Our results showed that the semantic information that is encoded in the HC features and the abstract-level information that are encoded in the TL and FT features contributed towards the selection of features in FF_Sig_. This finding implies that the association between FF_Sig_ and T stage occurred because the FF_Sig_ leveraged complementary information using both HC and deep ETs.

The FF_Sig_ determined a greater number of RRs with a greater number of genes when compared with the other image feature signatures. The FF_Sig_ was also correlated with EGFR. One potential explanation for our finding is that the FF_Sig_ encodes the imaging characteristics of the tumour that can reflect the underlying molecular characteristics of NSCLC^66^. The FF_Sig_ has also determined stronger inverse RRs with a range of genes when compared to HC_Sig_ and TL_Sig_. There was no stronger positive RRs with genes when compared with the HC_Sig_, TL_Sig_ and FT_Sig_. The reason for this is because the FF_Sig_ did not incorporate any image feature that was learned from scratch from the raw data using deep ETs; the FT components were the closest and as stated previously were aligned with the non-medical TL features. We suggest that positive RRs may appear when deep ETs are directly trained from scratch on the NRG-H CT data.

In addition, from our experiments using the NRG-H dataset, the FF_Sig_ determined RRs with a distinctive collection of GO terms with higher NES when compared to the other image feature signatures. A higher NES of GO terms is typically the result of a stronger correlation between the image feature signatures and the affiliated genes that contribute to the GO term and, RRs with a greater number of affiliated genes that contribute to the GO term. Notably, GO terms with the highest NES consist of a range of biological functions that relate to cellular structures. It has been reported that abnormalities in cellular structures are related to the development of NSCLC^67^. FF_Sig_ has shown to determine RRs with more GO terms when compared with HC_Sig_ and FT_Sig_. A potential explanation for this finding is that the FF_Sig_ determined RRs with a greater number of unique genes. These genes may be affiliated with a greater range of biological functions and therefore provide opportunities for FF_Sig_ to determine RRs with more and unique GO terms. We note that while the TL_Sig_ determined RRs with a higher number of GO terms, these are generally related to normal human anatomical information rather than the subtle disease processes related to the primary tumour. This finding is evidenced by the most enriched GO terms, such as “Regulation of Anatomical Structure Morphogenesis”, as shown in Table 3.

From our experiments using the NRG-H dataset, FF_Sig_ determined exclusive RRs with a group of GO terms that consist of a range of biological functions that are related to protein kinase activities, such as “Transmembrane Receptor Protein Kinase Activity”. Atypical kinase and its activities have been reported previously as an oncogene in NSCLC^68^, which play a crucial role in cell growth and tumourigenesis that may be observable in medical images^69^. In contrast, GO terms that are restricted to have RRs with FF_Sig_ include, for example, “Soluble Fraction” and “Enzyme Regulator Activity”. A potential explanation is that the specific enzyme activities and fractions cannot be depicted by CT images and hence cannot be quantified by the FF_Sig_.

Our validation experiments on the NRG-S dataset show that the FF_Sig_ comprises 13 image features that are complementary to image features that were selected in the HC_sig_, and FT_sig_. Among the 13 image features, 12 can be traced back to the 6144-dimensional FT features and the other feature can be traced back to a HC feature. Our results using NRG-S demonstrated that the FF_Sig_ encoded complementary medical imaging visual characteristics. The validation results are consistent with our previous findings from the NRG-H dataset.

However, none of the FF_Sig_, HC_Sig_, nor FT_Sig_ from the NRG-S dataset produced a significant association with the T stage. We attribute our findings to the different scanning parameters used in the NRG-S dataset, for example, slice thickness that ranges from 0.625 to 3 mm. Such factors contribute to subtle imaging differences and have potential impacts on the feature extraction process.

In our validation study, FF_Sig_ has determine a greater number of RRs with a greater number of genes when compared with the other image feature signatures. This result validates that the FF_Sig_ is robust and generalisable in encoding the imaging characteristics of the tumour that can reflect the underlying molecular characteristics of NSCLC. However, in our validation study, using the NRG-S dataset, FF_Sig_ did not identify stronger RRs with a range of genes when compared with HC_Sig_ and FT_Sig_. One potential explanation is that the FF_Sig_ did not incorporate any image feature that was fine-tuned on the NRG-S dataset. Despite NRG-S dataset has many similarities to the NRG-H dataset, such as the type of disease, the distribution of patients’ clinical parameters and their histopathology status are vastly different to the NRG-H dataset. We suggest that stronger RRs may appear when deep ETs are fine-tuned on the NRG-S dataset.

In our validation experiments, the FF_Sig_ has also shown to determine RRs with a distinctive collection of GO terms with higher NES when compared to the other image feature signatures. Notably, our validation results share a high degree of similarity with our previous findings from experiments using the NRG-H dataset. For example, from both experiments, the proposed FF_Sig_ determined RRs with GO terms such as ‘Membrane Enclosed Lumen’ and ‘Organelle Lumen’. Interestingly, such RRs with GO terms that relate to lumen structures are in opposite statistical direction. We attribute this finding to the differences between the NRG-H and NRG-S datasets where their distribution of T stage parameters and histology sub-types, as they played important roles in the multi-stage feature selection scheme. Such findings further demonstrate the robustness and generalisability of our proposed FF_Sig_ to determine RRs with GOs across different datasets.

Furthermore, in our validation experiments using the NRG-S dataset, FF_Sig_ determined exclusive RRs with a group of GO terms that consist of a range of biological functions that are related to peptidase activity such as ‘Endopeptidase Activity’. Previous study has shown that bombesin-like peptides and other neuropeptides are autocrine growth factors for both small cell lung cancer (SCLC) and NSCLC^70^. Our validation results demonstrate the robustness and generalisability of our proposed FF_Sig_ for determining GO terms that are related to NSCLC.

We recognise that a limitation of our study is the size of the dataset and that lack of knowledge about the patients’ mutation status. This limits the ability to optimise deep ETs to quantify image features that are most relevant to the NSCLC. Another limitation of this study is the differences between the train dataset and the independent test dataset. The two datasets use different methods for gene expression profiling, and as such the NRG-H dataset has a greater amount of genetic information compared to the NRG-S dataset. The ideal situation would have been to utilise two datasets that use the same technology for gene expression profiling, but at the time of experimentation and to the best of our knowledge, no such public radiogenomics dataset existed. However, despite these differences we note that the NRG-S dataset shares similarity with the NRG-H dataset, such as the type of disease and histopathology subtypes, and these similarities mean that it is the closest dataset that can be used for independent validation.

The limited availability of the clinical parameters e.g., survival data in the datasets has restricted our study from designing a deep learning-based image feature selection scheme. We note that as more radiogenomics datasets becomes available in the future, a key area for radiogenomics studies is to investigate the feasibility for a data-driven method for image feature selection^71^. Another potential future direction for our study is to investigate deep learning-based gene expression level prediction. Such a deep model can encode imaging characteristics that are reflective towards changes in gene expression levels and therefore may provide more insights into RRs.

## Conclusion

We used a selection of image features from handcrafted and deep ETs, which we named FF_Sig_, to determine RRs. Our results show that the FF_Sig_ encoded complementary medical image visual characteristics when compared with other image feature signatures. The FF_Sig_ determined more RRs with genes and with a group of distinct GO terms. Our results show that FF_Sig_ is correlated with a key biomarker for NSCLC and GO terms that are related to tumour developments in NSCLC. Furthermore, our validation experiments demonstrate that the FF_Sig_ is robust and generalisable in different dataset. The FF_Sig_ has demonstrated its potentials to identify important RRs that may facilitate cancer diagnosis and treatment in the future.

## Supplementary Information

Supplementary Information.
