# Supplementary material for: Fused feature signatures to probe tumour radiogenomics relationships
Source: Sci Rep. 2022 Feb 9;12:2173. doi: 10.1038/s41598-022-06085-y (PMC8828715; doi:10.1038/s41598-022-06085-y)
Supplement: Supplementary file 1 — Supplementary Information. [file 41598_2022_6085_MOESM1_ESM.docx]

Supplementary Materials

### 1. Tumour Delineation Confirmation Process

A second, independent clinician (7 years of experience) to confirm the region of interests (ROIs) that were delineated by the first clinician. The detailed procedure is as follows:

a. The second clinician first identified and localise the tumour and conducted delineation of ROIs, while being blinded to the clinical results and molecular status.

b. The second clinician then refined the localisations and delineation of ROIs, after the clinical results and molecular status were revealed to them.

c. The second clinician then confirmed the annotations defined by the first clinician by comparing their delineation of ROIs based on 1) tumour localisation and 2) shape and boundaries.

The second clinician was in strong agreement with the ROIs delineated by the first clinician in respect to 1) tumour localisation and 2) tumour shape and boundaries (Table S1). Based on this confirmation, we ran our experimental protocol with the original delineations by the first clinician.

### 2. Classification Performance Between Pre-trained Deep Models

We have assessed the potential for the following pre-trained models to serve as deep feature extraction techniques (ETs): Inception-V3^1^, VGG-19^2^, DenseNet-201^3^ and GoogLeNet^4^. The last layers of those pre-trained networks were replaced by a new fully connected layer to accommodate the classification task. The weight learn rate factor and bias learn rate factor were set to 20 for the fully connected layer. The pre-trained models were then fine-tuned by 1 epoch by using the training set of the NRG-H and their performances were accessed on the testing set of the NRG-H.

Table S6 compares the performance for the pre-trained models to classify images that contains tumours. Our results show that ResNet-101 outperformed most commonly used pre-trained models including Inception, GoogLeNet and DenseNet-201. VGG-19 outperformed ResNet-101, and hence it may be considered as an alternative pre-trained model for deep feature extractor (ET).

### 3. Supplementary Tables

**Table S1. Annotation Differences between the Two Clinicians.**

|  | Tumour localisation: Starting slice (z-axis) | Tumour localisation: Ending slice (z-axis) | Dice score |
| --- | --- | --- | --- |
| Annotation differences | 0.39 | 0.74 | 0.81 ± 0.01 |

**Table S2. Patient, Tumour and Histology Characteristics of the Non-small Cell Lung Cancer (NSCLC) Radiomics-Genomics Dataset (denoted as ‘NRG-H’ dataset in the manuscript) (n=89).**

| **Variable** | **Frequency (%)** |
| --- | --- |
| **Gender** |  |
| Male | 66.3 |
| Female | 33.7 |
| **T-stage** |  |
| T1 | 26.7 |
| T2 | 45.4 |
| T3 | 22.1 |
| T4 | 3.5 |
| N/A | 2.3 |
| **Histology sub-type** |  |
| Squamous cell carcinoma | 38.4 |
| Adenocarcinoma | 48.8 |
| Other | 12.8 |

**Table S3. Patient, Tumour and Histology Characteristics of the Non-small Cell Lung Cancer (NSCLC) Radiogenomics Dataset (denoted as ‘NRG-S’ dataset in the manuscript) (n=117).**

| **Variable** | **Frequency (%)** |
| --- | --- |
| **Gender** |  |
| Male | 75.2 |
| Female | 24.8 |
| **T-stage** |  |
| Tis | 4.3 |
| T1 | 44.4 |
| T2 | 37.6 |
| T3 | 9.4 |
| T4 | 4.3 |
| N/A | 0 |
| **Histology sub-type** |  |
| Squamous cell carcinoma | 22.2 |
| Adenocarcinoma | 75.2 |
| Other | 2.6 |

**Table S4. Comparison of Tumour Classification Results of Fine-Tuned Deep Model Across 5 Folds of the Training Set.**

| **Fine-tuning Cross- Validation** | **Validation Accuracy** | **Test Accuracy (n = 17)** | **Sensitivity** | **Specificity** | **Precision** | **f -measure** |
| --- | --- | --- | --- | --- | --- | --- |
| Fold 1 | 55.59% | 64.13% | 0.8396 | 0.5055 | 0.5377 | 0.6555 |
| Fold 2 | 58.64% | 61.52% | 0.7914 | 0.4945 | 0.5175 | 0.6258 |
| Fold 3 | 64.48% | 60.22% | 0.8503 | 0.4322 | 0.5064 | 0.6347 |
| Fold 4 | 63.68% | 64.78% | 0.9037 | 0.4725 | 0.5399 | 0.6760 |
| Fold 5 | 65.55% | 63.48% | 0.9144 | 0.4432 | 0.5294 | 0.6706 |

**Table S5. Tumour Classification Results of the Deep Model Fine-tuned on the NRG-H Dataset and Validated on the NRG-S Dataset.**

| **Fine-tuning Cross- Validation** | **Accuracy (n = 117)** | **Sensitivity** | **Specificity** | **Precision** | **f-measure** | **G mean** |
| --- | --- | --- | --- | --- | --- | --- |
| Fine-tuned Model | 49.9% | 0.8045 | 0.1930 | 0.4992 | 0.6161 | 0.3941 |

**Table S6. Comparison of the tumour classification performance between the pre-trained deep models on the testing set of the NRG-H.**

| **Pre-trained models** | **Accuracy (n = 17)** | **Sensitivity** | **Specificity** | **Precision** | **f-measure** | **G mean** |
| --- | --- | --- | --- | --- | --- | --- |
| VGG-19 | 72.34% | 0.95 | 0.50 | 0.65 | 0.77 | 0.69 |
| ResNet-101 | 68.86% | 0.84 | 0.54 | 0.65 | 0.73 | 0.67 |
| GoogLeNet | 63.55% | 0.90 | 0.37 | 0.59 | 0.71 | 0.58 |
| DenseNet-201 | 61.36% | 0.96 | 0.27 | 0.57 | 0.71 | 0.51 |
| Inception-V3 | 60.99% | 0.94 | 0.28 | 0.57 | 0.71 | 0.51 |

### 4. Handcrafted Features

We have extracted a total number of 431 handcrafted image features from CT volumes from the NSCLC dataset. The handcrafted features are defined by the Imaging Biomarker Standardisation initiative^5^ and can be sub-divided into the following groups:

1. First-order statistics
2. Shape and size based image features
3. Textural features
4. Wavelet features

### 4.1. First-order statistics

First-order statistics describes the distribution of voxel intensities within the tumour volume of interest (VOI). The following first-order statistics were extracted in the study:

1. Energy
2. Entropy
3. Kurtosis
4. Maximum
5. Mean
6. Mean absolute deviation
7. Median
8. Minimum
9. Range
10. Root mean square (RMS)
11. Skewness
12. Standard deviation
13. Uniformity
14. Variance

### 4.2. Shape and size based features

The following image features represent the three-dimensional size and shape of the tumour volume of interest (VOI). The following Shape and size based features were extracted in the study:

1. Compactness 1
2. Compactness 2
3. Maximum 3D diameter
4. Spherical disproportion
5. Sphericity
6. Surface area
7. Surface to volume ratio
8. Volume:

### 4.3. Textural features

**4.3.1 Gray-Level Co-Occurrence Matrix based features**

The Gray-Level Co-Occurrence Matrix (GLCM) based image features characterises the texture of the tumour region by using the histogram of co-occurring grayscale values at a given offset. The following GLCM based features were extracted in the study:

1. Autocorrelation
2. Cluster prominence
3. Cluster shade
4. Cluster tendency
5. Contrast
6. Correlation
7. Difference entropy
8. Dissimilarity
9. Energy
10. Entropy
11. Homogeneity 1
12. Homogeneity 2
13. Informational measure of correlation 1 (IMC1)
14. Informational measure of correlation 2 (IMC2)
15. Inverse Difference Moment Normalised (IDMN)
16. Inverse Difference Normalised (IDN)
17. Inverse variance
18. Maximum probability
19. Sum average
20. Sum entropy
21. Sum variance
22. Variance

**4.3.2 Gray-Level Run-Length matrix based features**

The Gray-Level Run-Length matrix (GLRL) based features quantifies the texture of the tumour region by characterising the number of consecutive pixels that have the same gray level values. The following GLRL based features were extracted in the study:

1. Short Run Emphasis (SRE)
2. Long Run Emphasis (LRE)
3. Gray Level Non-Uniformity (GLN)
4. Run Length Non-Uniformity (RLN)
5. Run Percentage (RP)
6. Low Gray Level Run Emphasis (LGLRE)
7. High Gray Level Run Emphasis (HGLRE)
8. Short Run Low Gray Level Emphasis (SRLGLE)
9. Short Run High Gray Level Emphasis (SRHGLE)
10. Long Run Low Gray Level Emphasis (LRLGLE)
11. Long Run High Gray Level Emphasis (LRHGLE)

### 4.4. Wavelet features: wavelet decompositions

Wavelet transformation allows the decomposition of images in low- and high-frequencies. Our experiment applied a discrete, one-level and undecimated three-dimensional wavelet transformation to each of the CT image volumes. The original CT image volume *X* was decomposed into eight decompositions. Let *L* and *H* denote the low- and high-pass function respectively. The resulted decompositions of *X* are labelled as *X_LLL_*, *X_LLH_*, *X_LHL_*, *X_LHH_*, *X_HLL_*, *X_HLH_*, *X_HHL_* and *X_HHH_*. In our experiment, wavelet “Coiflet 1” was applied to CT image volumes to derive all 8 decompositions. The first order statistics and textural features were computed for each of the decompositions.

### 5. Supplementary References

1 Szegedy, C., Vanhoucke, V., Ioffe, S., Shlens, J. & Wojna, Z. in *Proceedings of the IEEE conference on computer vision and pattern recognition.* 2818-2826.

2 Simonyan, K. & Zisserman, A. Very deep convolutional networks for large-scale image recognition. *arXiv preprint arXiv:1409.1556* (2014).

3 Huang, G., Liu, Z., Van Der Maaten, L. & Weinberger, K. Q. in *Proceedings of the IEEE conference on computer vision and pattern recognition.* 4700-4708.

4 Szegedy, C. *et al.* in *Proceedings of the IEEE conference on computer vision and pattern recognition.* 1-9.

5 Zwanenburg, A. *et al.* The image biomarker standardization initiative: standardized quantitative radiomics for high-throughput image-based phenotyping. *Radiology* **295**, 328-338 (2020).
